# Fuzzy obesity index (MAFOI) for obesity evaluation and bariatric surgery indication

**DOI:** 10.1186/1479-5876-9-134

**Published:** 2011-08-14

**Authors:** Susana Abe Miyahira, João Luiz Moreira Coutinho de Azevedo, Ernesto Araújo

**Affiliations:** 1 Universidade Federal de São Paulo (UNIFESP), Brazil. R. Botucatu 740 - São Paulo, SP, CEP 04023-900, Brazil; 2 Hospital Municipal Dr. José de Carvalho Florence (HMJCF), Av. Saigiro Nakamura 800 - São José dos Campos, SP, CEP 12220-280, Brazil; 3 Associação Paulista para o Desenvolvimento da Medicina (SPDM), Av. Saigiro Nakamura 800 - São José dos Campos, SP, CEP 12220-280, Brazil

## Abstract

**Background:**

The Miyahira-Araujo Fuzzy Obesity Index (MAFOI) for being used as an alternative in bariatric surgery indication (BSI) is validated in this paper. The search for a more accurate method to evaluate obesity and to indicate a better treatment is important in the world health context. Body mass index (BMI) is considered the main criteria for obesity treatment and BSI. Nevertheless, the fat excess related to the percentage of Body Fat (%BF) is actually the principal harmful factor in obesity disease that is usually neglected. The aim of this research is to validate a previous fuzzy mechanism by associating BMI with %BF that yields the Miyahira-Araujo Fuzzy Obesity Index (MAFOI) for obesity evaluation, classification, analysis, treatment, as well for better indication of surgical treatment.

**Methods:**

Seventy-two patients were evaluated for both BMI and %BF. The BMI and %BF classes are aggregated yielding a new index (MAFOI). The input linguistic variables are the BMI and %BF, and the output linguistic variable is employed an obesity classification with entirely new types of obesity in the fuzzy context, being used for BSI, as well.

**Results:**

There is gradual and smooth obesity classification and BSI criteria when using the Miyahira-Araujo Fuzzy Obesity Index (MAFOI), mainly if compared to BMI or %BF alone for dealing with obesity assessment, analysis, and treatment.

**Conclusion:**

The resulting fuzzy decision support system (MAFOI) becomes a feasible alternative for obesity classification and bariatric surgery indication.

## Background

The clinical conditions that are characterized as overweight (pre-obesity) and obesity are currently a universal epidemic of critical proportions. Efforts have been made to minimize this public health problem, but the prevalence of obesity is still growing in both developed and developing countries [1-6].

An excess of fat tissue (obesity) has been shown to be harmful for multiple organs and systems through trombogenic, atherogenic, oncogenic, hemodynamic, and neuro-humoral mechanisms [7-11]. Recently, obesity and related diseases (comorbidities), including *diabetes mellitus*, hypertension, coronary artery disease, cancer, sleep apnea, and osteoartrosis, have replaced tobacco use as a leading cause of death, where obesity contributes directly to the severity of the comorbities [12-15].

Therefore, a great clinical interest exists for evaluating overweight and obese patients to determine the risks inherent with these conditions, to prescribe and control conservative treatments, and to indicate when surgical treatment is needed. In the last 30 years, only the overweight and obesity rating system, which uses the body mass index (BMI), has been internationally recognized [16] (Table 1).

**Table 1:** Guidelines for the classification of overweight and obese adults using BMI

Condition	Classification	BMI
Overweight	OW	25 to 29.9
Obesity class I	OI	30 to 34.9
Obesity class II	OII	35 to 39.9
Obesity class III (Morbid)	OIII	≥40

Clinical guidelines on the identification, evaluation, and treatment of overweight and obesity in adults. Washington, National Institute of Health, 1998. (Modified).

BMI is a mechanism to measure weight excess extensively used in a myriad of epidemiologic studies, and is incorporated with clinical practice because of its simplicity [17]. However, it does not properly evaluate the body fat (BF) proportion because it fails to distinguish lean muscle mass from body fat [18]. The BF measurement has more value than global body mass measurements since the harmful factor in obesity is the accumulation of fat in the body, and lean muscle mass does not burden the individual health [19,20]. Additionally, the BMI itself is revealed as an imprecise and inaccurate method to measure the percentage of Body Fat (%BF), especially when people from different categories are took into account, which happens in populations of different ages and with different body types [21,22].

Despite of these limitations, the BMI is often used in the therapeutic approach to obesity classification, analysis, and treatment as well as to determine bariatric surgery (Table 2) [1].

**Table 2:** Indication of bariatric surgery according to the BMI and comorbidities

	BMI >35 and <40 Kg/m^2^	BMI >40 Kg/m^2^
Without comorbidities	Without indication	With indication
With comorbidities	With indication	With indication

Taking into account that the BF percentage is the most reliable indicator of obesity and that the BMI is used to prescribe surgery, it would also be convenient to simultaneously consider BF when approaching the patient to recommend bariatric surgery (Table 3) [23-25]. In this sense, the BMI should be included in conjunction with the %BF when evaluating the condition of the patient and determining an obesity treatment algorithm [18,26].

**Table 3:** Obesity classified by BF

BF (%)	Women	Men
ADEQUATE	<25%	<15%
LIGHT	25 - 30%	15 - 20%
MODERATE	30 - 35%	20 - 25%
HIGH	35 - 40%	25 - 30%
MORBID	>40%	>30%

Guideline for the classification of obesity in adults. National Institute of Diabetes and Digestive and Kidney Diseases. U.S. Department of Health and Human Services. (Modified).

Therefore, the search for a more accurate model that evaluates overweight and obese patients with apparent body mass excess led to the conception that indicates when surgery is appropriate for these patients. Previously presented, the Miyahira-Araujo Fuzzy Obesity Index (MAFOI) evaluates the obesity by correlating BMI and the BF in the context of fuzzy set theory and fuzzy logic. MAFOI must also have the ability to accurately recommend which patients should be referred for bariatric surgery.

## Objectives

General: To determine a more accurate parameter for the evaluation of obesity and in bariatric surgical indication.

Specifics:

1) To evaluate the use of Miyahira-Araujo Fuzzy Obesity Index (MAFOI) in a random sample of the obese population.

2) To validate Miyahira-Araujo Fuzzy Obesity Index (MAFOI) in indicating bariatric surgery.

## Methods

This prospective study was carried out at the Hospital Municipal Dr. José de Carvalho Florence (HMJCF), in the city of São José dos Campos, São Paulo state, Brazil from December of 2008 to August of 2009. Such a research is approved by the Ethic and Research Commission (CEP) of the Universidade de Taubaté (UNITAU) (Exhibit I) and the Universidade Federal de São Paulo (UNIFESP) (Exhibit II). All participants in the study signed an informed consent form that was in accordance with Decree no. 196/96 of the National Health Council (CNS)/Health Ministry (MS) and its complements (Decrees 240/97, 251/97, 292/99, 303/00, and 304/00 of the CNS/MS) (Exhibit III). This research was sponsored by the funding agency Fundação de Amparo à Pesquisa do Estado de São Paulo (FAPESP), process # 2009/07956-7.

Inclusion criteria were the following: patients from emergency and nursing rooms in the HMJCF, of both gender, and aged 18 years and older, and patients fasting at least for 6 hours of solid food and 4 hours of liquids. Exclusion criteria were the following: patients who refused to take part in the study, pregnant women, and patients with kidney failure, hydroelectrical alterations, inadequate hydration, fever (T>37.8°C), ascites, hepatic cirrhosis, a coronary by-pass, or an amputation of the inferior or superior members.

The weight, height, and BF of the patients were measured during the same day and at subsequent time points.

### BMI Calculation

To calculate the BMI, a stadiometer, which was graded at every 0.5 cm, and a digital scale, with 0.1-kg sensitivity, were used.

### BF Calculation

To obtain BF and fat-free mass (FFM) values, a body composition analyzer was used, a method that uses direct multi-frequency bio-impedance (BIA) and the Segmental-model InBody230 (Biospace Co., Ltd. Seoul 135-784 KOREA) Tetra-polar System with 8-points. The BF values and FFM system were obtained through the BIA from equations that were incorporated in the equipment, as described by Bedogni [35].

#### Protocol for the evaluation

1) The patients were instructed to refrain from drinking alcohol and to not perform heavy physical activity during the day prior to the exam.

2) Fasting at least for 6 h of solid food and 4 h of liquids prior to the exam.

3) The patients were instructed to use the rest room before the test.

4) The patients wore light clothes or a hospital gown.

5) The patients did not wear watches or jewelry in the vicinity of the electrodes.

6) The patients remained standing for 5 minutes before the exam performance.

7) The room temperature at the exam was maintained from 20°C to 25°C.

#### Fuzzy Set Theory and Fuzzy Logic for Fuzzy BMI, Fuzzy %BF and Fuzzy Obesity Output Classes and Values in Obesity Assessment

**Figure 1:**
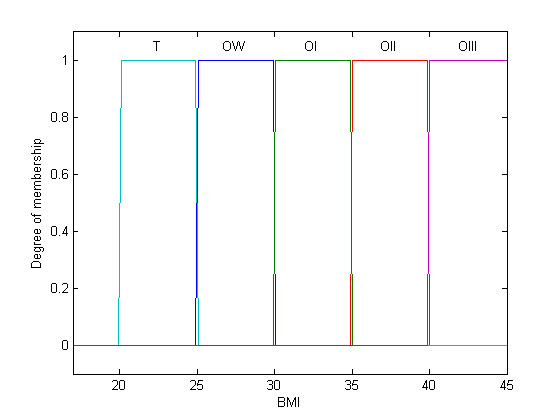
**Classical BMI**. BMI classical set, with the linguistic values: slim (S), overweight (OW), obesity class I (OI), obesity class II (OII), obesity class III (OIII).

**Figure 2:**
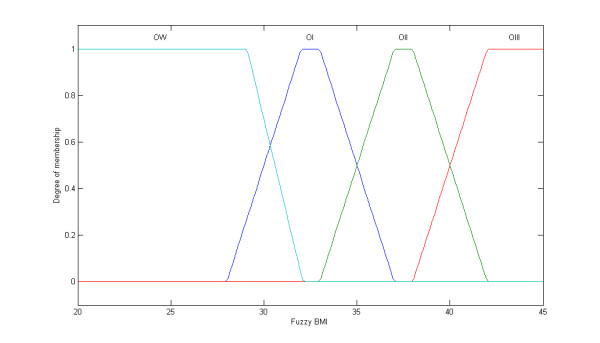
**Fuzzy BMI**. BMI fuzzy set, with the linguistic terms: overweight (OW), obesity class I (OI), obesity class II (OII), obesity class III (OIII).

**Figure 3:**
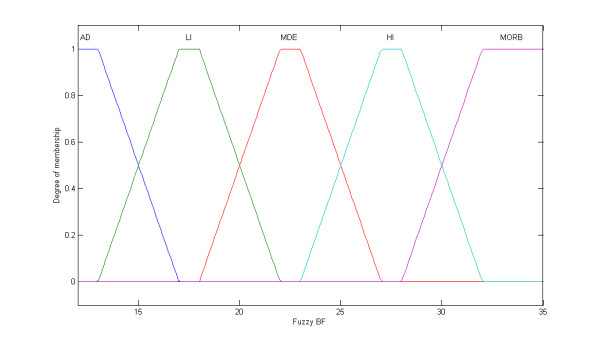
**Fuzzy BF**. BF fuzzy set, with the linguistic terms: adequate (AD), light obesity (LI), moderate obesity (MDE), high obesity (HI), morbid obesity (MORB).

**Figure 4:**
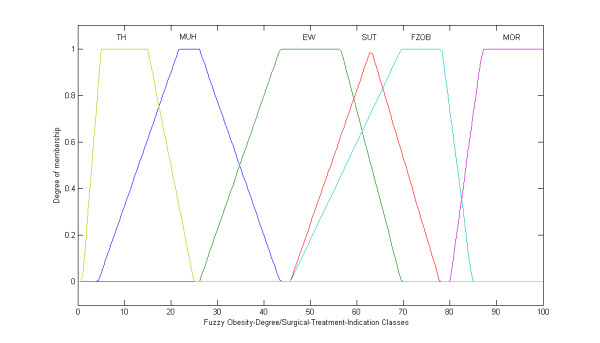
**Fuzzy Obesity-Degree/Surgical-Treatment-Indication Classes**. Obesity-Degree/Surgical-Treatment-Indication classes set, with the linguistic terms: thin (TH), muscular hypertrophy (MUH), excess of weight (EW), sutomori (SUT), fuzzy obesity (FZOB), and morbid obesity (MOR).

Initially, the BMI was modified by the treatment of the crisp classes, as adopted by the World Health Organization (WHO), into fuzzy sets, i.e., fuzzy classes (Figure 1 and 2). While the classical set theory is based on the excluded middle principle where an element belongs, or not, to a set (crisp set/class), the fuzzy set theory allows a relation of gradual membership of an element to a determined set [27,28]. Such an approach was, thus, extended to the %BF classes (Figure 3). The fuzzy BMI and fuzzy %BF classes were aggregated by employing logical connectives and mapped into fuzzy obesity output classes and values resulting in a new index named the Miyahira-Araujo Fuzzy Obesity Index (MAFOI) (Figure 4). MAFOI was, then, used to classify individuals in relation to their obesity condition and establish a criterion that provides a decision-making system that can recommend bariatric surgery, as well.

These described steps embrace the mapping process that includes the following: (*i*) the knowledge basis, (*ii*) the fuzzification that translates the crisp value (classical number) of the input variable into a fuzzy value, (*iii*) the cylindrical extension, the aggregation, the conjunction, and the projection, and (*iv*) the *defuzzification *that translates the output linguistic variable in a crisp value.

To build the input variable for the fuzzy BMI, the WHO classification (Table 1) was used. The fuzzy sets for the fuzzy BMI are assigned the following linguistic terms: overweight (OW), obesity class I (OI), obesity class II (OII), and obesity class III (OIII).

To build the input variable for the fuzzy %BF, the NIDDK classification of overweight and obesity was used (Table 3). The fuzzy sets for the fuzzy %BF are assigned the following linguistic terms: adequate (AD), light obesity (LI), moderate obesity (MDE), high obesity (HI), and morbid obesity (MOR).

The fuzzy obesity or surgical-treatment-indication evaluation constituted the output linguistic variable (consequent of the rule). The fuzzy sets for the fuzzy obesity or surgical-treatment indication are assigned the following linguistic terms: thin (TH), muscular hypertrophy (MUH), excess of weight (EW), sutomori (SUT), fuzzy obesity (FZOB), and morbid obesity (MOR). The rules were restricted to those classes considered relevant, i.e. restricted to only those than can happen in ordinary practice (Table 4).

**Table 4:** Bases of Fuzzy Rules

BMI/BF	TH	OW	OI	OII	OIII
**AD**	TH	MUH	MUH	MUH	X
LI	TH	HM	HM	HM	X
**MDE**	EW	EW	SUT	SUT	MOR
**HI**	EW	FZOB	FZOB	FZOB	MOR
**MOR**	X	FZOB	FZOB	FZOB	MOR

BMI (body mass index), overweight (OW), obesity class I (OI), obesity class II (OII), and obesity class III (OIII). BF (body fat percentage), adequate (AD), light obesity (LI), moderate obesity (MDE), high obesity (HI), thin (TH), muscular hypertrophy (MUH), excess of weight (EW), sutomori (SUT), fuzzy obesity (FZOB), and morbid obesity (MOR).

The base of rules is represented as a fuzzy matrix in table 4.

#### Fuzzy BMI, % Fuzzy BF, Fuzzy Obesity Output Classes, and MAFOI performance to obesity diagnosis and to surgical treatment indication

The WHO reference standard is employed to evaluate the obesity diagnosis performance, which is evaluated by using the BMI (Table 1). Values that are already described in the literature were used to evaluate the obesity-diagnosis performance, which was evaluated using the %BF cut-off value [25]. To evaluate the MAFOI, a value defined by the *defuzzification *of the output variable is used by using the center of area method.

### Statistical analysis

The continuous variables are presented as mean and standard deviation (SD) and numbers and percentages as categorical variables. The Pearson coefficients of correlation and the respective intervals of confidence (IC) (95%) are estimated to compare BMI, BF and MAFOI by genre. The McNemar test [29] is used to compare the percentage of the individuals considered obese by the BMI versus BF, BMI versus MAFOI and BF and BF versus MAFOI.

## Results

In the current study, 81 patients were evaluated and 72 out of the 81 were evaluated by analyzing the BMI and %BF. Among the excluded patients, 7 were not fasting, a patient had consumed alcohol within 24 h prior to the test, and a patient had a fever (T = 38.2°C) at the time of evaluation. Within the 72 patients, 42 were female and 30 were male. The mean age standard deviation (SD) was 39.5 ± 11.2 years old for women and 43.5 ± 15.8 years old for men. The mean weight SD was 70.0 ± 14.5 kg for women and 79.6 ± 25.3 kg for men. The mean BMI SD was 27.1 ± 5.8 kg/m^2 ^for women and 27 ± 7.4 kg/m^2 ^for men. The mean %BF SD was 38.7 ± 6.7% for women and 26.3 ± 7.9% for men. The demographic data are described in Table 5.

**Table 5:** Standard deviation (SD), body mass index (BMI), body fat (BF)

	Women (n = 42)				Men (n = 30)			
	**Mean**	**Minimum**	**Maximum**	**SD**	**Mean**	**Minimum**	**Maximum**	**SD**

**Age** (years)	39.5	18.0	60.0	11.2	43.5	18.0	76.0	15.8
**Weight** (Kg)	70.0	48.0	113.1	14.5	79.6	32.0	160.0	25.3
**Height** (m)	160.9	148.5	170.0	5.7	172.2	155.5	183.0	7.5
**BMI** (Kg/m^2^)	**27.1**	**18.8**	**45.9**	5.8	**27.0**	**17.6**	**54.1**	7.4
**BF** (%)	**38.7**	**25.2**	**48.8**	6.7	**26.3**	**9.9**	**40.1**	7.9

The maximum and minimum BMI, %BF, and MAFOI values are presented in Table 6. Mean and SD values are given for BMI and %BF. Table 7 displays the Pearson linear correlation coefficients between BMI (Kg/m^2^) and the remaining variables: BF, FFM, and MAFOI for both genders.

**Table 6:** Standard deviation (SD), body mass index (BMI), body fat (BF)

	Women (n = 42)				Men (n = 30)			
	**Mean**	**Minimum**	**Maximum**	**SD**	**Mean **	**Minimum**	**Maximum**	**SD**

**BMI**	**27.1**	**18.8**	**45.9**	5.8	**27.0**	**17.6**	**54.1**	7.4
**BF **(%)	**38.7**	**25.2**	**48.8**	6.7	**26.3**	**9.9**	**40.1**	7.9
**MAFOI**		**23.9**	**91.7**			**23.9**	**91.7**	

The maximum and minimum BMI, BF, and MAFOI values.

**Table 7:** Body mass index (BMI), body fat (BF), fat free mass (FFM)

		Women (n = 42)	Men (n = 30)
**BMI and BF**	Pearson correlation	0.831	0.656
	Sig. (2-tailed)	<0.001	<0.001
**BMI and FFM**	Pearson correlation	0.683	0.848
	Sig. (2-tailed)	0.000	<0.001
**BMI and MAFOI **	Pearson correlation	0.770	0.617
	Sig. (2-tailed)	<0.001	<0.001
**BF and MAFOI**	Pearson correlation	0.905	0.961
	Sig. (2-tailed)	<0.001	<0.001

The Pearson linear correlation coefficients between BMI (Kg/m^2^), BF (%), FFM (Kg), and MAFOI for both genders.

The low bound value of BMI obesity class I classification (OI) = 30 and the low bound value of %BF high obesity classification (HI) = 35 (women = 35; men = 25+10), which are defined by the WHO/NIDDK [16,25] were used as input values of the fuzzy model. The fuzzy inference was performed. The outcome was the cut-off value of index MAFOI/BSI (MAFOI) = 68.

The percentage of individuals that were considered obese by the BF criteria was statistically lower than by the BMI criteria (Table 8).

**Table 8:** Body mass index (BMI), body fat (BF)

**BF**			
>35(women) >25(men)			
**BMI** >30 kg/m^2^	OBESE	NON-OBESE	
OBESE	16	1	17 (23.6%)
NON-OBESE	30	25	55
TOTAL	46 (63.9%)	26	72

The percentage of individuals considered obese by the BF and the BMI criteria.

The percentage of obese individuals determined by the MAFOI criteria was statistically higher than by the BMI criteria (Table 9). The percentage of obese individuals determined by the BF criteria was statistically higher than the MAFOI criteria (Table 10).

**Table 9:** Body mass index (BMI)

**MAFOI**			
>68			
**BMI** >30 kg/m	OBESE	NON-OBESE	
OBESE	12	5	17 (23.6%)
NON-OBESE	18	37	55
TOTAL	30 (41.7%)	42	72

The percentage of individuals considered obese by the MAFOI and the BMI criteria.

**Table 10:** Body fat percentage (%BF)

**MAFOI**			
**>68**			
**BF**			
>25 men	OBESE	NON-OBESE	
>35 women			
OBESE	30	16	46 (63.9%)
NON-OBESE	-	26	26
TOTAL	30 (41.7%)	42	72

The percentage of individuals considered obese by the MAFOI and the BF criteria.

The correlation between the BMI and %BF for women was stronger than for men. When comparing BMI to FFM, the correlation was better for men. The groups show a strong correlation for all of the variables in both genders. Regarding the BMI and MAFOI, the correlation was strong for both women and men. The correlation between BF and MAFOI was the best one for both genders.

The percentages of individuals that were considered obese by the BMI, %BF, and MAFOI criteria are presented in Table 11. The percentage of individuals considered obese by the %BF criteria (63.9%) was statistically higher than the BMI criteria (23.9%) (p < 0.001). The percentage of individuals considered obese by the MAFOI criteria (41.7%) was statistically higher than the BMI criteria (23.6%) (p < 0.001). The percentage of individuals considered obese by the %BF criteria (63.9%) was statistically higher than the MAFOI criteria (41.7%) (p < 0.001) [30].

**Table 11:** Body mass index (BMI), body fat (BF)

**BMI = 23.6%**	**BF = 63.9%**
>30	>35(women)
	>25(men)
**BMI = 23.6% **	**MAFOI = 41.7%**
>30	>68
**BF - 63.9%**	**MAFOI = 41.7%**
>35 (women)	>68
>25(men)	
n = 72	

The percentages of individuals that were considered obese by the BMI, BF, and MAFOI criteria.

## Discussion

### Use of BMI to classify obesity

Despite its limitations, the BMI is currently considered the most useful measurement of the obesity level of the population. Thus, the BMI can be used to estimate the prevalence of obesity in the population and the risks associated with this condition. However, it does not elucidate the wide variation in the nature of obesity between different individuals and diverse populations.

Among sedentary and overfed individuals, the increase of body mass is generally due to both body fat and muscle mass. Nevertheless, among men, the increase of body mass may play a more important role than in women which has the increase of body fat the main factor of acquired excess of weight. Thus, the correlation between the BMI and %BF for women is stronger than for men. When comparing BMI to fat-free mass, the correlation was better for men, a feasible explanation is due to the greater increase of the muscle mass among them. Regarding the BMI and MAFOI, the correlation was strong for both men and women. The correlation between BF and MAFOI was the best one for both genders.

Studies indicate that the BMI has to be adjusted for diverse ethnical groups as the WHO study of the Western Pacific Region [31]. This study demonstrated that different cut-off values must be adapted for overweight (>23 kg/m^2^) and for obesity (>25 kg/m^2^). Other studies evaluated the Australian aborigine population and showed that the cut-off point was >26 kg/m^2 ^for defining overweight [31]. The BMI accuracy in diagnosing obesity is mainly limited in intermediary ranges of BMI in men and in elders due to a failure in discriminating free-fat mass and body fat [32].

The results of this study were in agreement with the data found in the literature when the performances of the BMI and BF in diagnosing obesity were compared [18,32,33]. Analyzing only the BMI, 23% of the sample was considered obese, while this proportion increased to 63.9% and 41.7% when evaluated, respectively, with the %BF and the MAFOI.

The variability between living things of the same species, inherent to the biological condition, allows a range of classification. However, the limits of these artificially created classes are inaccurate and badly defined.

To justify the use of fuzzy logic in this research, it is worth to consider that the classical procedure for evaluating the results from research in the life-science area has been the application of descriptive statistics to the tabulation and stratification of data. Inferential statistics have been used where probabilistic analyses are needed. In the classical logic approach, however, all of the instruments aim at establishing values with a higher rate of occurrence; specific ranges of variables are directly defined as causes or modulating factors. This treatment is perfectly suited when it refers to results of exact-science studies where the objects are simple substances and the samples are homogeneous. However, this is not the case in the biological field where the disparity observed can be simply due to normal individual variation that occurs in a species population [34].

### Limitations of the study

1) The membership functions were conceived by the authors based on the concepts, classification and knowledge about overweight and obesity already described in the literature [25]. Therefore others membership functions maybe acceptable. 2) The fact that there is not a MAFOI for men and other for women. The only one obtained maybe creates a skewness that underestimates BSI for men as the BF cut-off for men may be considered. 3) The calculus of the MAFOI itself was decided taking into account the lower bounds of two special bands of BMI and %BF categorization. This election seems adequate since those special bands include the obese subjects, however studies may continue to analyze clinical conditions like metabolic syndrome, hypertension, and cancer. 4) The rules appear to be reasonable, since they are building up based on the logical concept. 5) The accurateness of all the assumptions adopted for the fuzzy inference system can be verified according to the matching against real data where BSI had been achieved as a good decision. Finally, the development carried out in this paper admits other representations since it allows subtle changes, modifications in the output can be verified.

## Conclusion

The Miyahira-Araujo Fuzzy Obesity Index (MAFOI) demonstrated to be adequate both to evaluate the obesity condition and to recommend bariatric surgery according to experimental data.

The MAFOI results are closer to the real clinical condition of obesity of the individual than either the BMI or the %BF.

## Competing interests

The authors declare that they have no competing interests.

## Authors' contributions

SAM made an extensive research on the bibliography, and was the responsible for the data collection. JLMCA designed the study in a methodological point of view, and was the principal writer of this study in English. EA was the responsible for the fuzzy logic approach. All authors read and approved the final manuscript.

## Appendix

**MAFOI: Fuzzy Set Theory, Fuzzy Logic building Fuzzy Obesity Assessment according to Fuzzy BMI, Fuzzy %BF, and Fuzzy Output Classes and Values **[26,37]:

The fuzzy set theory and fuzzy logic can be understood both as a manner to reproduce the knowledge and the common sense working as an interface between numbers and symbols (linguistic expression) as a tool to build up numerical functions when dealing with data [36,37].

The concept underlying *fuzzy sets *allows the gradual and not absolute pertinence from an element to a class, contrary to the classical sets. A *classic set*, *M*, in a space of points assigned *universe of discourse*, *X *= {*x*}, is defined by a *characteristic function*, *μ_M_*(*x*), that assumes a null value for all elements of *X *that not belongs to the set *M*, *μ_M_*(*x*) = 0 if x ∉ M, and a unitary value for those values that belong to it, *μ_M_*(*x*) = 1 if x ∈ M, i.e., *μ_M_*(*x*): *X *→ {0, 1}. Differently, a *fuzzy set*, *M*, in a universe of discourse, *X*, is defined by a *membership function*, *μ_M_*(*x*): *X *→ [0, 1]. If the values of *μ_M_*(*x*) are, in turn, associate to a degree of truthiness, the truth is assigned to continuous values within [0, 1] [27,28]. The membership function *μ_M_*(*x*) can also be understood as the compatibility degree among fuzzy sets which, in turn, are related to linguistic terms.

(1) The first step for achieving the Miyahira-Araujo Obesity Index (MAFOI) is, thus, accomplished when the BMI is modified into fuzzy sets by the treatment of the crisp classes adopted by the World Health Organization (WHO), as depicted in Figure 1 and 2[26]. To build the input variable for the BMI, the WHO classification in Table 1 is used. In sequence, such a process is extended to %BF classes (Figure 3) [26]. To build the input variable for the %BF, the NIDDK classification of overweight and obesity in Table 3 is used. The elements of BMI and the elements of %BF, both being distributed into the universes of discourses *X *and *Y*, respectively, are grouped and assigned by classes or linguistic terms. The BMI obesity classes are assigned the linguist terms *overweight *(OW), *obese class I *(OI), *obese class II *(OII), and *obese class III *(OIII) meanwhile the %BF obesity classes are assigned the linguistic terms *adequate *(AD), *light obesity *(LI), *moderate obesity *(MDE), *high obesity *(HI), *morbid obesity *(MOR) [26].

When employing the classical set theory to classify obesity and to recommend surgical treatments, or not, there is categorical, crisp classes like *yes *or *no*, *recommendation *or *no-recommendation for bariatric surgery*. Diverse crisp obesity classes can be employed for surgical recommendation, according to the class a patient belongs to (Figure 1). For instance, a patient with a BMI of *39 *Kg/m^2 ^is assigned to the *Obesity II class*, such that *μ_M = OII _*(*x = 39 *Kg/m^2^) = *1*. Observe that all the other classes obtain a null activation status, *μ_≠OII _*(*x = 39 *Kg/m^2^) = *0*. This category achieves *no-recommendation *class for bariatric surgery, *μ_no-recommendation _*(*x = *39 Kg/m^2^) = *1*, or equally null surgical recommendation, *μ_recommendation _*(*x = *39 Kg/m^2^) = *0 *[37]. Nevertheless, it seems to be arbitrary to assign a Boolean approach as the one used for BMI or %BF. Two patients with BMI of 39 kg/m^2 ^and BMI of 40 kg/m^2 ^are, respectively, classified into the OII and OIII groups receiving each a distinct treatment recommendations, even if the difference from one patient to the other is minimal, Δ1. Although the first patient is not in the range for a surgical recommendation, the second one is in the range for a surgical recommendation. In this situation, both patients may not present significant biological, anatomical, or physiopathological differences that justify such a discrepancy in the surgical recommendation. Conversely, fuzzy set theory allows simultaneously allocating a patient in more than one class, or not, by embodying the inherent subjectivity in the obesity and bariatric surgery classification and analysis processes. Likewise crisp obesity classification, fuzzy obesity classification also allows dealing with diverse groups and classes (Figure 2). This provides the advantage of a more realistic classification both for obesity severity and surgical recommendations. Taking into account the same patient, a fuzzy set (class) assigned *Obesity II Class *is active with a degree of *recommendation *- i.e., a degree of certainty - for surgical treatment, *μ_recommendation_^OBII ^*(*x = *39 Kg/m^2^) = *α*_1_, where 0 <*α*_1 _< 1, due to a degree of membership, *μ_M = OBII _*(*x = *39 Kg/m^2^) = *α*_1_. Observe that this patient may also be classified by another fuzzy set labeled *Obesity III Class *achieving another degree of *recommendation *for surgical treatment, *μ_recommendation_^OBIII ^*(*x = *39 Kg/m^2^) = *α*_2_, where 0 <*α*_2 _< 1, according to a different degree of membership, *μ_M = OBIII _*(*x = *39 Kg/m^2^) = *α*_2_, such that *α*_1 _*> α*_2 _[37]. Further, when taking into account two patients with BMI of 39 kg/m^2 ^and BMI of 40 kg/m^2^, both would be categorized either as OII as OIII. The difference exists since the first patient presents a class of OII that is higher than OIII, whereas the second patient is more in the OIII group than in the OII group. In this case, both patients have a potential to receive or not receive a recommendation for surgical treatment. This determination depends on other factors and not only the BMI value, which is improperly and perhaps inconsistently used.

(2) The second step in building up the MAFOI is fulfilled by satisfying the BMI dependence upon another factor [26]. Fuzzy set theory advantages in allowing distinct variables to work together based on the aggregation of their respective fuzzy sets. The manipulation of sets is chiefly carried out by operators of intersection ∩, union ∪, and complement, ¬. The intersection set operation corresponds in logic to the connective, operator of conjunction, ⋀, and to the semantic connective, "and" The union set operation is associated to the connective operator of disjunction, ⋁, and to the semantic connective "or" The complement is related to the logical connective of negation of a given proposition presenting the idea of opposition. The BMI and %BF classes were aggregated by employing logical connective of conjunction. The %BF variable is the modulation factor for BMI variable in the obesity degree and surgical recommendation analysis. When the sets are considered under the classical set theory, the Cartesian pair, (*x*,*y*), such that *x *∈ BMI and *y *∈ %BF, assumes either a unitary value, *μ*(*_M_^BMI ^*× *_M_^%BF^*) (*x,y*) = 1, for each pair that belongs to the relationship or a null value, *μ*(*_M_^BMI ^*× *_M_^%BF^*) (*x,y*) = 0, for each pair that does not belong to the relationship. When the partition of the universe of discourse for the BMI and %BF variables is accomplished by using the fuzzy set theory, each Cartesian pair is also able to assume an intermediary value between 0 and 1, 0 *μ*(*_M_^BMI^× _M_^%BF^*) (*x,y *1, yielding an overlapping of classes (overlapped assignments) in a way that the patient can be classified in complementary manners. Both BMI and %BF are understood as input variable when dealing with a fuzzy IF-THEN inference mechanism (mapping) and the resulting Cartesian product, *X *× *Y*, is related to the input space. In general, this input space is mapped into an output universe of discourse.

(3) This leads to the third step in designing the Miyahira-Araujo Fuzzy Obesity Index. The obesity-degree/surgical-treatment-indication evaluation constituted the output linguistic variable (Figure 4) [26]. The fuzzy sets that part such an output universe of discourse are assigned the linguistic terms *thin *(TH), *muscular hypertrophy *(MUH), *excess of weight *(EW), *sumotori *(SUT), *fuzzy obesity *(FZOB), and *morbid obesity *(MOR). They were obtained according to the classification of body composition, regarding the weight, muscle mass, and body fat. The sutomori fuzzy set for obesity is also a novel obesity class previously introduced by the authors and there is no similar in literature.^26 ^It is a special body constitution which is found among sumo wrestlers, characterized by a large amount of both muscles and fat tissue. These athletes have a large muscular mass and present a high level of %BF and due to that are usually considered as obese. However, when compared with individuals with equivalent BMI, they present lower values of %BF [26].

(4) The fourth and latter step for obtaining the MAFOI is related to its proper structure that maps the BMI and %BF linguistic variables into the obesity-degree/surgical-treatment-indication linguistic variable by employing the fuzzy logic [26]. Fuzzy logic is essentially a system of rules of inference characterized as a set of (IF-THEN) rules. This mechanism of fuzzy inference uses logic principles to establish how facts and rules have to be combined to derive new facts. An important concept is the *fuzzy rules*, IF *P_1 _*AND *P_2 _*AND ... AND *P_n _*THEN *Q *where the set of input fuzzy propositions, *P_i _*= *x_i _*is *M_i_*, *i *= 1, ..., *n*, and the inferred fuzzy proposition, *Q *= *z *is *Ni*, are called, respectively, *premises *(antecedent of the rule) and *conclusion *(consequent of the rule) such that the fuzzy rules can also be represented as IF *x_1 _*is *M_1j _*AND *x_2 _*is *M_2j _*AND ... AND *x_n _*is *M_nj _*THEN *z *is *Ni*. Being a mechanism of inference, the fuzzy logic is understood as a form to represent the human approximate reasoning; being a form to represent a mapping, it is a universal approximator.^36,37 ^The rules were restricted to those considered relevant; i.e., they were restricted to feasible rule than can really occur in real health world. Given the set of fuzzy IF-THEN rules as established in Table 4 the Miyahira-Araujo Fuzzy Obesity Index is, then, used to classify individuals in relation to their obesity condition and establish a criterion that provides a decision-making system that can recommend bariatric surgery [26].

## References

[B1] Kolata G Obesity declared a disease Science 1985 227 1019 20 10.1126/science.3975599 3975599

[B2] Bray GA The epidemic of obesity - A chronic disease that governments worldwide must take seriously West J Med 2000 172 78 9 10.1136/ewjm.172.2.78 10693361 PMC1070754

[B3] Haslam DW James WPT Obesity Lancet 2005 366 1197 209 10.1016/S0140-6736(05)67483-1 16198769

[B4] James PT Leach R Kalamara E Shayeghi M The worldwide obesity epidemic Obes Res 2001 9 228S 233S 10.1038/oby.2001.123 11707546

[B5] Fine JT Colditz GA Coakley EA Moseley G Manson JAE Willett WC Kawachi I A prospective study of weight change and health-related quality of life in women JAMA 1999 282 2136 42 10.1001/jama.282.22.2136 10591335

[B6] Abelson P Kennedy D The obesity epidemic Science 2004 304 1413 8 10.1126/science.304.5676.1413 15178768

[B7] Hampton T Scientists study fat as endocrine organ JAMA 2009 296 1573 5 10.1001/jama.296.13.1573 17018794

[B8] Lago F Gómez R Gómez-Reino JJ Dieguez C Gualillo O Adipokines as novel modulators of lipid metabolism Trends Biochem Sci 2009 3 500 10 10.1016/j.tibs.2009.06.008 19729309

[B9] Wozniak SE Gee LL Watchel MS Frezza EE Adipose Tissue: The New Endocrine Organ? A Review Article Dig Dis Sci 2009 54 1847 56 10.1007/s10620-008-0585-3 19052866

[B10] Nathan C Epidemic inflammation: pondering obesity Mol Med 2008 14 485 92 18431463 10.2119/2008-00038.Nathan PMC2323335

[B11] Wellen KE Hotamisligil GS Inflammation, stress, and diabetes J Clin Invest 2005 115 1111 19 15864338 10.1172/JCI25102 PMC1087185

[B12] Visscher TLS Seidell JC Menotti A Blackburn H Nissinen A Feskens EJM Kromhout D Underweight and overweight in relation to mortality among men aged 40-59 and 50-69 years Am J Epidemiol 2000 151 660 6 10752793 10.1093/oxfordjournals.aje.a010260

[B13] WHO Obesity: preventing and managing the global epidemic. Report of a WHO consultation 2000 894 World Health Organ Tech Rep Ser i xii 1-253 11234459

[B14] McLachlan CR Poulton R Car G Cowan J Filsell S Greene JM Taylor DR Welch D Williamson A Sears MR Hancox RJ Adiposity, asthma, and airway inflammation J Allergy Clin Immunol 2007 119 634 9 10.1016/j.jaci.2006.10.029 17141852

[B15] Geloneze B Mancini MC Coutinho W Obesity: knowledge, care, and, commitment, but not yet cure Arq Bras Endocrinol Metabol 2009 53 117 119 10.1590/S0004-27302009000200001 19466202

[B16] Calle EE Thun MJ Petrelli JM Body-mass index and mortality in a prospective cohort of U.S. adults N Engl J Med 1999 341 1097 105 10.1056/NEJM199910073411501 10511607

[B17] Eknoyan G Adolphe Quetelet (1796-1874)--the average man and indices of obesity Nephrol Dial Transplant 2008 23 47 51 17890752 10.1093/ndt/gfm517

[B18] Okorodudu DO Jumean MF Montori VM Romero-Corral A Somers VK Erwin PJ Lopez-Jimenez F Diagnostic performance of body mass index to identify obesity as defined by body adiposity: a systematic review and meta-analysis Int J Obes 2010 34 791 9 10.1038/ijo.2010.5 20125098

[B19] Adams TD Heath EM LaMonte MJ Gress RE Pendleton R Strong M Smith SC Hunt SC The relationship between body mass index and per cent body fat in the severely obese Diabetes Obes Metab 2007 9 498 505 10.1111/j.1463-1326.2006.00631.x 17587392

[B20] Liu A McLaughlin T Liu T Sherman A Yee G Tsao PS Differential Intra-abdominal Adipose Tissue Profiling in Obese, Insulin-resistant Women Obes Surg 2009 19 1564 73 10.1007/s11695-009-9949-9 19711137 PMC3181138

[B21] Jackson AS Ellis KJ McFarlin BK Sailors MH Bray MS Body mass index bias in defining obesity of diverse young adults: the Training Intervention and Genetics of Exercise Response (TIGER) Study Br J Nutr 2009 102 1084 90 10.1017/S0007114509325738 19344545 PMC2873180

[B22] Razak F Anand SS Shannon H Vuksan V Davis B Jacobs R Teo KK McQueen M Yusuf S Defining Obesity Cut Points in a Multiethnic Population Circulation 2007 115 2111 8 10.1161/CIRCULATIONAHA.106.635011 17420343

[B23] Lee JW Wang W Lee YC Huang MT Ser KH Chen JC Effect of laparoscopic mini-gastric bypass for type 2 diabetes mellitus: comparison of BMI>35 and <35 kg/m2 J Gastrointest Surg 2008 12 945 52 10.1007/s11605-007-0319-4 17940829

[B24] Staub K Ruhli FJ Woitek U Pfister V BMI distribution/social stratification in Swiss conscripts from 1875 to present Eur J Clin Nutr 2010 64 335 40 10.1038/ejcn.2010.7 20160753

[B25] National Institute of Diabetes and Digestive and Kidney Diseases Understanding adult obesity 1993 NIH- Publ. n° 94-3680. Rockvilli, MD: National Institute of Health

[B26] Miyahira SA Araujo E Fuzzy obesity index for obesity treatment and surgical indication IEEE International conference on fuzzy systems (Fuzz-IEEE). Hong Kong 2008 2392 7

[B27] Zadeh LA Fuzzy control Informat Control 1965 8 338 53 10.1016/S0019-9958(65)90241-X

[B28] Zadeh LA Probability measures and fuzzy events J Math Anal Appl 1968 23 421 7 10.1016/0022-247X(68)90078-4

[B29] Eliasziw M Donner A Application of the McNemar test to non-independent matched pair data 2006 10.1002/sim.4780101211 1805322

[B30] World Health Organization Western Pacific Region. The Asia-Pacific perSDective: Redefining obesity and its treatment 2000 WHO

[B31] Romero-Corral A Somers VK Sierra-Johnson J Thomas RJ Collazo-Clavell ML Korinek J Allison TG Batsis JA Sert-Kuniyoshi FH Lopez-Jimenez F Accuracy of body mass index in diagnosing obesity in the adult general population Int J Obes 2008 32 959 66 10.1038/ijo.2008.11 PMC2877506 18283284

[B32] Waisbren E Rosen H Bader AM Lipsitz SR Rogers SO Eriksson E Percent Body Fat and Prediction of Surgical Site Infection J Am Coll Surg 2010 210 381 9 10.1016/j.jamcollsurg.2010.01.004 20347729

[B33] Pan WH Yeh WT How to define obesity? Evidence-based multiple action points for public awareness, screening, and treatment: an extension of Asian-Pacific recommendations Asia Pac J Clin Nutr 2008 17 370 4 18818155

[B34] Seising R From vagueness in medical thought to the foundations of fuzzy reasoning in medical diagnosis Artificial Intelligence in Medicine 2006 38 237 5 10.1016/j.artmed.2006.06.004 16956755

[B35] Bedogni G Malavoti M Severi S Poli M MUssi C Fantuzzi AF Battisni N Accuracy of an eight-point tactile-electrode impedance method in the assessment of total body water Eur J Clin Nutr 2002 56 1143 8 10.1038/sj.ejcn.1601466 12428182

[B36] Araujo E Fuzzy Logic and Approximate Reasoning: Concepts and Application Synergismus scyentifica 2009 04 2 1 16

[B37] Miyahira SA Araujo E Miyahira-Araujo Fuzzy Obesity Index (MAFOI) for Body Mass Index and Body Fat Clinical Analysis, Syndrome Assessment, Classification and Treatment, and Surgical Indication IEEE Trans on Fuzzy Systems 2011 in press

